# Earlier Flowering of *Betula pendula* Roth in Augsburg, Germany, Due to Higher Temperature, NO_2_ and Urbanity, and Relationship with *Betula* spp. Pollen Season

**DOI:** 10.3390/ijerph181910325

**Published:** 2021-09-30

**Authors:** Franziska Kolek, Maria Del Pilar Plaza, Vivien Leier-Wirtz, Arne Friedmann, Claudia Traidl-Hoffmann, Athanasios Damialis

**Affiliations:** 1 Department of Environmental Medicine, Faculty of Medicine, University of Augsburg, 86156 Augsburg, Germany; franziska.kolek@tum.de (F.K.); maria.plaza@tum.de (M.D.P.P.); vivien.leier-wirtz@tum.de (V.L.-W.); claudia.traidl-hoffmann@tum.de (C.T.-H.); 2 Faculty of Applied Computer Sciences, Institute of Geography, University of Augsburg, 86159 Augsburg, Germany; arne.friedmann@geo.uni-augsburg.de; 3 Helmholtz Center Munich, German Research Center for Environmental Health, Institute of Environmental Medicine, 86156 Augsburg, Germany; 4 Christine Kühne Center for Allergy Research and Education (CK-CARE), 7265 Davos, Switzerland; 5 Department of Ecology, School of Biology, Aristotle University of Thessaloniki, 54124 Thessaloniki, Greece

**Keywords:** flowering phenology, nitrogen dioxide, pollen allergy, temperature, urbanity

## Abstract

Flowering and pollen seasons are sensitive to environmental variability and are considered climate change indicators. However, it has not been concluded to what extent flowering phenology is indeed reflected in airborne pollen season locally. The aim of this study was to investigate, for the commonly represented in temperate climates and with highly allergenic pollen *Betula pendula* Roth, the responsiveness of flowering to different environmental regimes and also to check for commensurate changes in the respective pollen seasons. The region of Augsburg, Bavaria, Germany, was initially screened for birch trees, which were geolocated at a radius of 25 km. Random trees across the city were then investigated during three full flowering years, 2015–2017. Flowering observations were made 3–7 times a week, from flower differentiation to flower desiccation, in a total of 43 plant individuals. Data were regressed against meteorological parameters and air pollutant levels in an attempt to identify the driving factors of flowering onset and offset. Flowering dates were compared with dates of the related airborne pollen seasons per taxon; airborne pollen monitoring took place daily using a Hirst-type volumetric sampler. The salient finding was that flowering occurred earlier during warmer years; it also started earlier at locations with higher urbanity, and peaked and ended earlier at sites with higher NO_2_ concentrations. Airborne pollen season of *Betula* spp. frequently did not coincide locally with the flowering period of *Betula pendula*: while flowering and pollen season were synchronized particularly in their onset, local flowering phenology alone could explain only 57.3% of the pollen season variability. This raises questions about the relationship between flowering times and airborne pollen seasons and on the rather underestimated role of the long-distance transport of pollen.

## 1. Introduction

Allergic diseases comprise a major health problem, affecting up to 40% of the population of Europe [1,2,3]. They have been increasing in prevalence and are expected to further increase in the future up to 50% of the European population [4]. This rise has been particularly pronounced in pollen allergies. As an example, the sensitisation rate in Germany in 1998 was 29.8% and rose to 33.6% in 2008–2011 [5].

The above rates may further increase if one takes into account additional environmental parameters, such as air quality. Worldwide, nine out of 10 people are affected by air pollution [6]. It is known that air pollutants pose negative effects on human health and can cause or promote the development of respiratory diseases [7], with this influence being more pronounced in children [8]. Exposure to air pollutants also increases the risk of developing an allergy or of worsening allergic symptoms [9].

Allergic patients can have a significantly worsened life quality due to their allergy symptoms [10]. This reduction of life quality can be caused by a negative impact on social activities, lower quality of sleep, or reduced overall performance [11]. Apart from the serious effects on the personal life of allergic individuals, pollen allergies also exhibit a considerable economic burden for the health system due to school and work absenteeism [3,11,12].

While the exact mechanisms responsible for the rising frequency and severity of respiratory allergies are not completely understood, it has been already documented that the westernised lifestyle and air pollution do affect the prevalence of allergic symptoms [13]. Since plants have been characterised as sensitive indicators for climate change, they act as reliable proxies for monitoring changes in the environment (their pollen included) [14,15]. The influence of environmental factors (e.g., meteorology, air pollution, biodiversity, urbanity) particularly on anemophilous plants is frequently reflected in changes in the seasonality of their airborne pollen [16,17] and can give an estimate on future scenarios of climate variability [18]. Therefore, the research in the current study attempts to address the occurrence (presence), abundance (concentration), and timing (phenology) of airborne pollen in Augsburg, Germany.

Moreover, plant phenology has been often reported to be connected with changing environmental conditions. Factors like precipitation, soil humidity, air pollutants and competition are known to influence the timing of phenology [19,20]. Here, the observation of the development of male inflorescences and especially the flowering period of different individuals of *B. pendula* in the study region is thematised. Temporal and spatial patterns influencing the timing of phenological observations were emphasised upon. Therefore, it has been considered timely and important to continuously record airborne pollen to be able to observe longer-term changes in their biodiversity and relative abundance patterns. Such changes and responses are expected to be more pronounced in sites like the study area of Augsburg, which is situated close to the German Alps. It has been documented that high mountain regions are especially influenced by rising temperatures and alterations in precipitation [21], which concomitantly may lead to biodiversity changes. It is already known that the study area has experienced, between 1961 and 2017, significant increasing trends in yearly temperatures, while at the same time yearly precipitation has decreased [22].

Given the high allergenicity of *Betula* pollen [23] and the complete lack of any information on the biodiversity and abundance of airborne pollen in Augsburg, Germany, the present work is important and novel providing an environmental health service for the citizens and visitors of the study area. Along with aerobiological monitoring, phenological observations help to characterise the timing and intensity of flowering of anemophilous plants and add a valuable spatial component to the standard aerobiological monitoring. Such information from plant individuals may significantly contribute to the identification and to the ecological interpretation of specific favourable environmental conditions of plant growth and stress tolerance. This sensitivity of flowering to different environmental regimes is the one that to date has led to reliable predictions of crop, fruit and seed production levels [24]. Finally, from a public health perspective, quantification of the flowering season occurrence for plants that produce allergenic pollen contributes to improved allergy management practices [25,26]. Overall, flowering (and consequently airborne pollen) has been declared a sensitive bio-indicator of climate change by the Intergovernmental Panel on Climate Change (IPCC) and both the observations of plant phenology in general and airborne pollen monitoring have been comprising valuable tools for the detection, quantification and prediction of environmental change [27,28,29,30].

Having said the above, the here presented work attempts to investigate airborne pollen transport and flowering phenology as well as the connection between these processes and their interaction with the ambient environment, for *Betula* spp., being a widespread genus in temperate climates [31,32], and its pollen comprising one of the major allergy triggers in Europe.

## 2. Materials and Methods

The study area of the presented work consists of the region in and around the city of Augsburg (48°36′ N, 10°89′ E, 494 m above sea level), which is situated in Bavaria, in the south of Germany. The area lies north of the Alps on an outwash plain of the post ice age, influenced by the Alpine rivers, Lech, Wertach and Singold merging in Augsburg.

The climate is temperate oceanic (Köppen climate classification Cfb; Temperate, without dry season, warm summer) with a yearly mean temperature of 13.2 °C and a mean total precipitation of 766 mm (30 year average, 1981–2017) [22]. It belongs to the biogeographical region “continental” [33]. The highest precipitation is measured in July (99.7 mm) and the lowest in February (36.6 mm).

**Figure 1:**
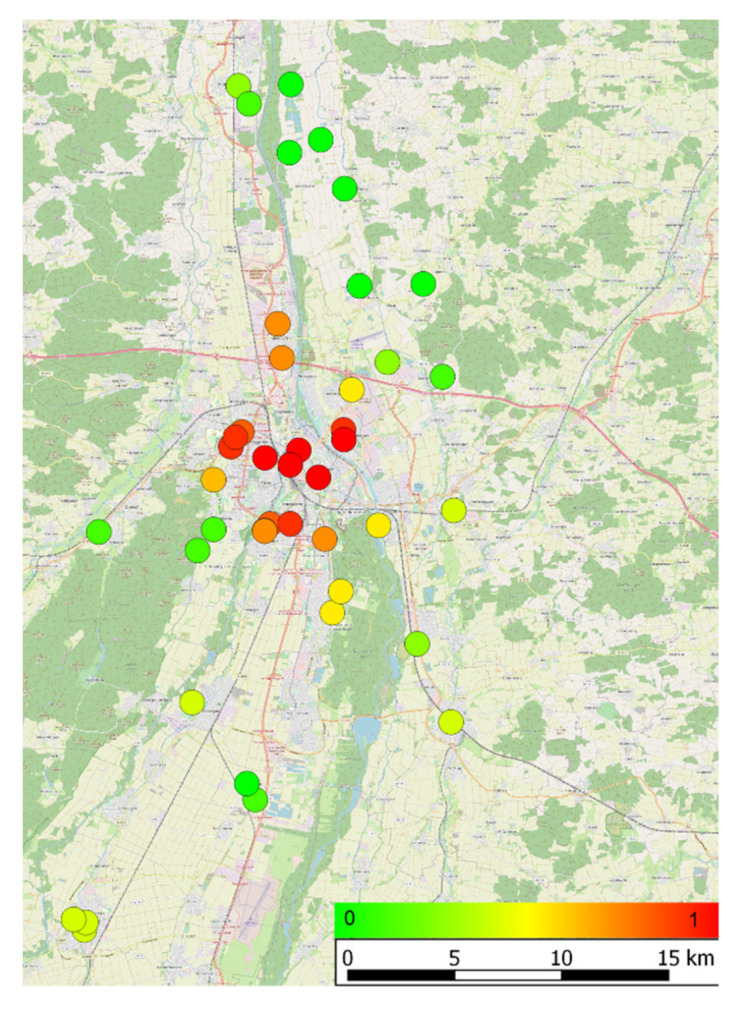
Location and Urbanity Index (UI) for all observed *Betula pendula* individual trees. (0: green (rural), 1: red (urban).

The aerobiological sampling site is located in the southern part of the city of Augsburg at 48.326078 N, 10.903089 E, 496 m above sea level. To study the flowering phenology, individual trees were selected in a radius of 25 km around the city of Augsburg (Figure 1). For the phenological observations, 43 individual trees were sampled and observed between 2015 and 2017. These were randomly selected after screening for *Betula* species across the region of Augsburg. In total, 5622 individual *Betula* plants were geolocated, out of which 5380 (95.7%) belonged to *B. pendula*. The exact location of each tree was assessed with a Garmin Dakota 10 GPS device (Garmin Deutschland GmbH, Garching, Germany) in the World Geodetic System WGS 84. The morphometric traits of the trees were also assessed, namely tree height, crown height and diameter, and trunk perimeter, for every observed tree. The tree height was measured from the ground to the highest point of the crown, using an optical hand clinometer by Breithaupt (F. W. Breithaupt & Sohn, Kassel, Germany). The crown height was measured with the same technique from the lowest branch of the tree to the highest point of the crown. The trunk perimeter was measured in a height of 1.00 m–1.20 m with a measuring tape at a height of the trunk without branches or excrescences. For the crown diameter, the distance from the trunk to the most distant point of the crown was measured in four directions with a measuring tape. The mean of these values was calculated. 

### 2.1. Environmental Factors

#### 2.1.1. Urbanity Index

The urbanity index (UI) used in this study was assessed for every and each observed tree according to the method developed by Jochner et al. [34]. The index is based on the CORINE 2006 land cover data [35] and describes the part of urban areas in an area of 2 km around each tree. When the index is 0, this refers to a rural environment with no urban areas, whereas an index of 1 describes an urban environment without any non-urban areas. Instead of using more generalised indices of vegetation or land cover, we used the specific radius of the 2-km surroundings per individual tree; thus, mesoclimatic effects are taken into account so as to accurately identify either intensively urbanised areas or roughly urban ones [34]. The index was assessed using QGIS Version 2.4.0 (QGIS Development Team (2018). QGIS Geographic Information System. Open Source Geospatial Foundation Project http://qgis.osgeo.org (accessed on 15 February 2021). 

#### 2.1.2. Air Quality Measurements

Air quality measurements were performed in all three study years at all observed trees between March and April, in parallel to the phenological observations and the sampling of pollen. The measurements were conducted by use of special devices, namely passive samplers, attached on each tree and obtaining individualised microscale measurements in-situ (the analysis of the passive samplers was performed by passam ag, Zurich, Switzerland).

#### 2.1.3. Meteorological Parameters

Hourly data for temperature and precipitation were collected via the German Weather Service (Deutscher Wetterdienst (DWD), Offenbach, Germany 2019). The weather station (DWD ID: 232) is situated at Augsburg Airport (48.4254; 10.9420) in the north of the city of Augsburg, on an elevation of 461 m above sea level. Cumulative temperature was assessed and processed, from 1 January: we used the accumulated temperature, as pollen and flowering seasons are influenced not only by the immediate temperature, but also by the lagged temperature values just before flowering [36]. As it is known that minimum temperature has been frequently the limiting factor in phenological development [37], the cumulative temperatures were calculated based on the daily minimum values.

#### 2.1.4. Pedological and Geological Parameters

Pedological and geological aspects for all observed sites were assessed from the BayernAtlas, a service for geographical maps in Bavaria, provided by the Bayerische Vermessungsverwaltung. For pedology, the soil type [38] was extracted from the “Übersichtsbodenkarte” (overview soil map) [39] and characterized afterwards by main grain size and potential nutrient availability. The main grain size is an indicator for potential water availability, as it is known that a higher amount of medium to coarse material in the soil reduces the soil water availability for plants [40]. The information about the geology was extracted from the geological map [39] and categorised afterwards by the main grain size as an indicator of potential water availability.

The variables were pooled in different categories. For the soil, grain sizes are categorised as fine (silt and clay; grain size < 0.036 mm), medium (sand; grain size 0.036 mm–2 mm) and coarse (gravel, cobble; grain size > 2 mm) [41]. Mixed grain sizes with similar proportions of different grain sizes were categorised as mixed. Areas in the city with no pedological data were categorised as unknown. The potential water and nutrient availability was categorised as high, noderate, low, following the description in the pedological map [39]. Soil types with inconsistent features were marked as diverse. For the parent material, the grain sizes were categorised as for the soil as fine (silt and clay; grain size < 0.036 mm), medium (sand; grain size 0.036 mm–2 mm) and coarse (gravel, cobble; grain size > 2 mm) [41].

#### 2.1.5. Surroundings

In addition to the above-mentioned environmental factors, the surrounding environment of the tree also plays an important role. Not just the soil and geology, but also the surface of the soil, the vegetation close to the tree, and the shading caused by natural or artificial elements. The surface of the soil can influence the water availability, for example, by being sealed, compressed, or covered with different kinds of vegetation. The vegetation close to the tree can influence the availability of water and nutrients, whereas shading can result in a lower energy input and lower temperatures for the tree. The above refer to competition for more sunlight, more water, and more nutrients.

The surroundings of each tree were assessed in a radius of 5 m, as it is known that the roots of *Betula pendula* can reach a diameter of up to 6 m [42]. This area was characterised with regards to the sealing of the surface, the type of the ground cover and the appearance and type of different plants. Therefore, the following parameters were investigated (Table 1).

### 2.2. Aerobiological Monitoring in Augsburg

The sampler is located on ground level, so the sample is taken from a height of 1.6 m which resembles the position of the human upper airway to reflect the exposure of a human to the collected particles. Aerobiological monitoring abides by the international standard, as defined by the European Aerobiology Society [42]. In brief, in Augsburg, since 2015, the full diversity of airborne pollen (and fungal spores) has been monitored with a Hirst-type volumetric trap (Burkard Manufacturing Co. Ltd., Uxbridge, UK) [43]. Pollen measurements were performed between the end of March to the end of October for all three years in the study. The air samples were analysed with a Leica DM750 light microscope (Leica Mikrosysteme Vertrieb GmbH, Wetzlar, Germany). With a 400× magnification, pollen of 45 different taxa were identified and counted on a bi-hourly basis. Pollen counts were then converted and expressed into concentrations and expressed as pollen grains per m^3^ of air. The main pollen season was defined according to Nilsson and Persson [44], as the 5% (onset) and 95% (end) of the cumulative sum of the annual pollen season. 

### 2.3. Phenological Flowering Observations of Betula Pendula

**Figure 2:**
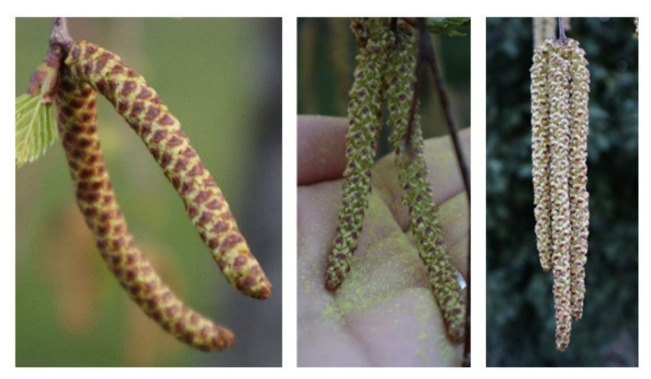
Flowering phenophases of *Betula pendula*. Left: closed inflorescences, middle: open inflorescence with prominent stamens (start of flowering), right: desiccated inflorescence without stamens (end of flowering).

The BBCH (Biologische Bundesanstalt für Land- und Forstwirtschaft, Bundessortenamt und Chemische Industrie) developed a scale to code similar growth stages from different plants [45]. This scale was confirmed by the COST Action 725 on a European level [46]. The relevant stages for the development of the flowers in the male inflorescences, and therefore relevant for the described observations, are the principal growth stages 5 and 6. These stages were observed for every tree in every year for 6 weeks from mid-March to the end of April, every second day, from stages 51 to 55 and stages 65 to 69. For the stages 59 to 64, the observations were performed daily. The main phenophases, especially those related to pollen liberation and dispersion, are shown in Figure 2.

As not all male inflorescences are synchronised in the same stage of floral development during each observation, the catkins were observed randomly each time on a defined area of the crown. To have stable, comparable results for all observations, a frame of 50 cm × 50 cm was used for observing the male inflorescences in this area. The frame was positioned on random places on the crown in every observation to guarantee a non-biased result. All male inflorescences within this frame and in a depth of 50 cm were counted per development stage (winter stage, pre-flowering, start of flowering, full flowering, end of flowering, male inflorescences falling). The numbers of inflorescences per development stage were noted afterwards. 

### 2.4. Data Analysis

The phenological characteristics of flowering were examined, specifically those connected with pollen emission, viz. the start, peak, end, and duration of flowering. Differences among individuals and sampling sites in the phenological traits were checked using ANOVA and post-hoc Bonferroni test, as well as explorative techniques, like hierarchical cluster analysis (Ward’s clustering). When testing for additional, continuous co-factors, like meteorological parameters or air pollutants, full-factorial analysis of variance and covariance were applied (ANOVA, ANCOVA). The relationships between phenological characteristics of flowering and growth traits of individuals were also investigated, using the full set of data and performing simple and full factorial regressions. The flowering dates per individual *Betula pendula* tree and per year were checked against the pollen season dates of the respective pollen season of *Betula* spp. over the same year of study. All aerobiological data were checked against flowering phenological data for lag effects (GLM, time series analysis, cross-correlations). The correlation coefficients and the specific lags at the significance level *p* < 0.05 were defined per year. Based on statistically significant differences deriving from the above-mentioned tests, and particularly focusing on the interaction effects of multiple independent variables, ridge regressions were conducted per a dependent variable and for all studied parameters, namely flowering and pollen season attributes. Ridge regressions are well-known for dealing with multi-collinearity issues, and partial correlations aid in identifying the most significant parameters and their lag effects and synergistic effects among independent variables, as well as confounding factors. For all analyses described above and for the visualisation of results, Box-Whisker plots were used for showing differences among individuals or between years. Last, scatterplots (with linear) regression fits were applied with the respective confidence intervals to express significant slopes and quantify the respective effects.

All analyses were run at the significance level of *p* = 0.05 and by use of Statistica^TM^ (TIBCO Software Inc., Palo Alto, CA, USA) Version 13.3 or Microsoft^®^ Excel^®^ (Microsoft Corporation, Redmond, WA, USA) 2016. To assess the data distribution and normality, Kolmogorov-Smirnov tests were run, and residual analyses were conducted per dataset and separate analysis. In most cases, data were normally or log-normally distributed and logarithmic transformations were also tested and applied where appropriate.

## 3. Results

**Figure 3:**
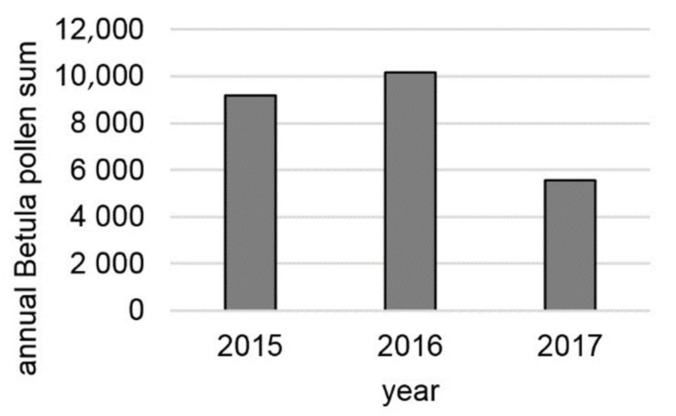
The annual pollen integrals of *Betula* spp. in Augsburg, during 2015–2017.

The amount of airborne *Betula* pollen differs between years, with the annual pollen integral being statistically lower, particularly in 2017 (Figure 3).

**Figure 4:**
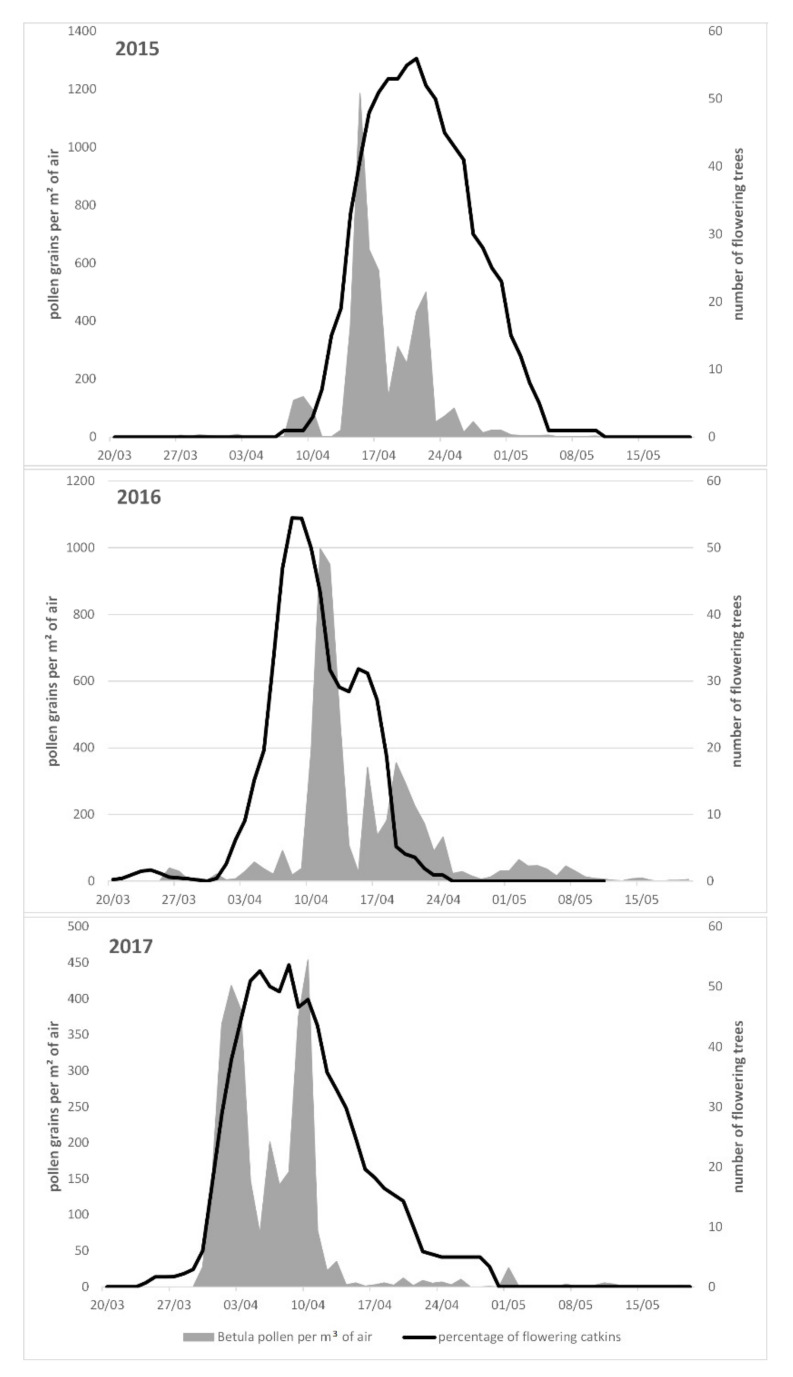
Seasonal patterns of pollen atmospheric circulation for *Betula* spp. and flowering phenology patterns for *Betula pendula* that is the main representative of this pollen taxon in the study area. The *X* axis corresponds to the observation date; the *Y* axis (left) corresponds to the mean percentage of open inflorescences per individual plant and the *Y*’ axis (right) to the pollen atmospheric concentration (per m^3^ of air). The grey area indicates airborne pollen concentrations, whereas the black line phenological observations of flowering. Based on time-series analyses (cross-correlations), all pairs of seasons significantly correlate between them (*p* < 0.001).

Regarding the *Betula* main pollen season, this was calculated as 5–95% of the annual amount of pollen [44], and during 2015–2017 it lasted for 16 days in 2015 (9–24 April), for 26 days in 2016 (7 April–2 May), and for 14 days in 2017 (1–12 April) (Figure 4, *Y* axis, grey area). On average, the *Betula* pollen season takes place from 2 April until 25 April and lasts 24 days, with the peak occurring on 15 April.

Considering the flowering phenology of *Betula pendula*, as may be seen in Figure 4 (*Y*’ axis, black line), the earliest start of flowering was observed on 28 March, in 2017, the latest end of flowering on 30 April, in 2015. The average start of the flowering for all observed individuals in all years was observed on 10 April (±6 days), while the end was on average on 18 April (±7 days). The average duration of the flowering season was 10.3 ± 3.7 days.

**Figure 5:**
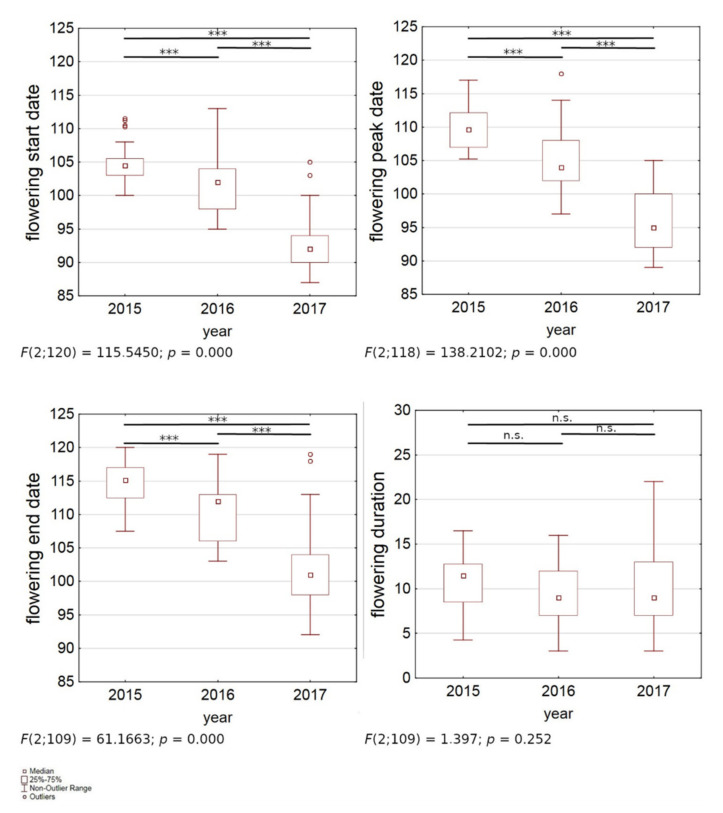
Differences between years in *Betula pendula* flowering season (*Y* axis: start, peak, end dates, and duration) in Augsburg during 2015–2017 (*X* axis) (ANOVA, ns: not significant relationship, ***: *p* < 0.001).

The flowering start date, peak date and end day of the observed *B. pendula* trees differed between the different years of observation. The latest start of flowering was observed in 2015 (on average: 15 April), the earliest start, being 12 days earlier than 2015, was observed in 2017 (on average: 3 April). (Figure 4 and Figure 5). It is noticeable that the average flowering duration does not significantly differ between the years (Figure 5). It is evident that when the flowering season starts earlier on a year, it also ends earlier, with the seasons being shifted, instead of prolonged. 

When comparing the pollen season of *Betula* spp. vs. the flowering season of the most representative *Betula pendula* in Augsburg, it can be seen that the seasons are generally coinciding concerning their timing but show significant differences in the shape of the season. In 2015 and 2016, there were days on which pollen were measured in the air but none of the observed trees was flowering yet. And also there were days in all three observed years when there were trees observed flowering but the amount of pollen in the air was low compared to the number of flowering trees (Figure 4).

**Figure 6:**
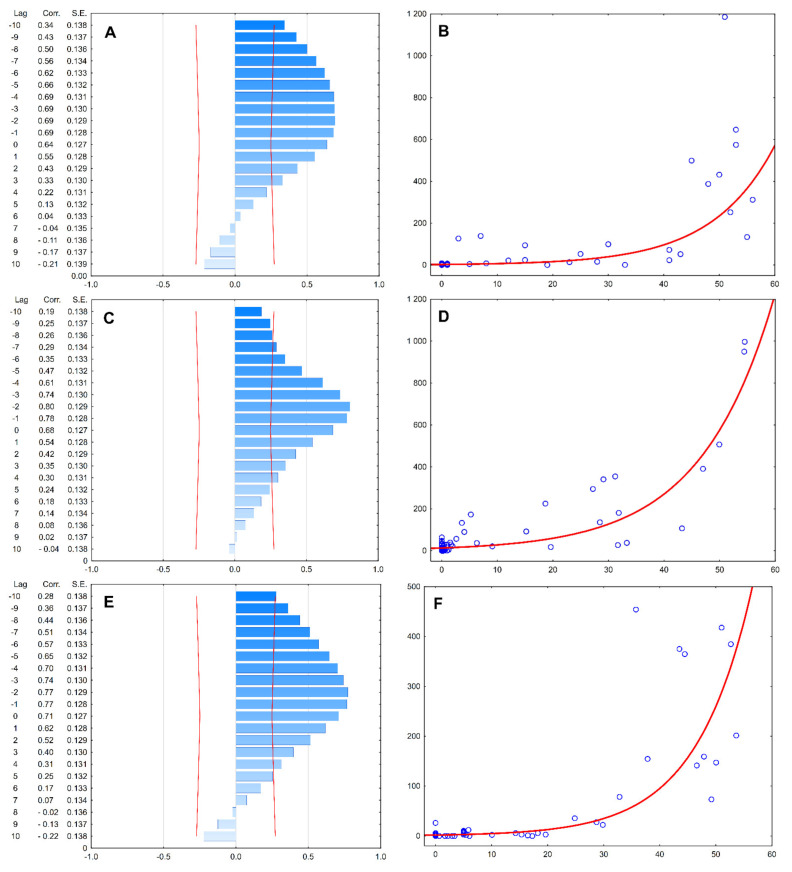
Relationships between pollen season (dependent variable) and flowering season (lagged variable) in Augsburg, during 2015–2017 (time-series analysis, cross-correlations): (**A**,**B**): 2015, (**C**,**D**): 2016, (**E**,**F**): 2017.

It can be concluded that pollen and flowering seasons are cross-correlated, with the pollen season being influenced by the flowering of up to 10 days before (Figure 6A,C,E). The strongest correlation was consistently observed with a delay effect of −2 days for the pollen season, with the relationship between the flowering and pollen season being exponential, for the trees and the time period studied (Figure 6B,D,F). Nevertheless, the magnitude of the effect was varying within the three years of study, from *R*^2^ = 0.48 in 2015, up to 0.64 and 0.60 during 2016 and 2017, respectively, but being steadily low (Figure 6A,C,E). Overall, the *Betula* spp. pollen seasons may be explained by the *B. pendula* season locally by only 57.3% (Figure 6).

The left panel shows the cross-correlations for a range of −10 to +10 lags (red line marks the confidence interval at *p* = 0.05).

The right panel shows the relationship (simple regression with exponential fit, at the strongest correlation lag for all years (−2)) between the pollen concentration of *Betula* spp. (*Y* axis) vs. the number (%) of flowering catkins of *Betula pendula* trees (red line marks the regression fit at confidence interval *p* = 0.05).

**Figure 7:**
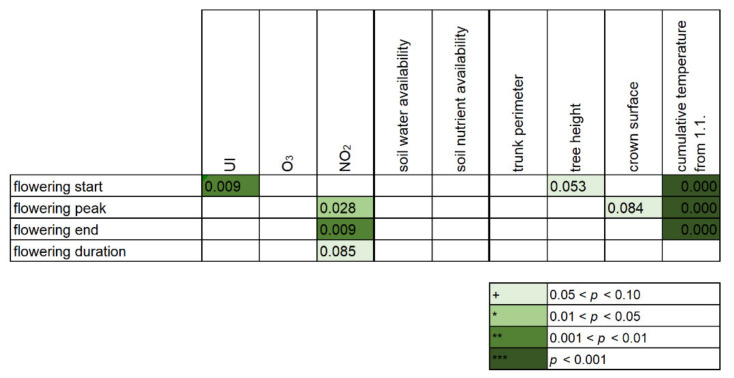
Heatmap showing the relationships of flowering season traits (onset, peak, end and duration) against various environmental parameters (ridge regression, backwards stepwise elimination).

As far as the most significant factors influencing the flowering season in Augsburg are concerned, air temperature was proven to be the most prominent environmental parameter to consistently determine the phenological traits of flowering as evidenced by the backwards stepwise ridge regression (Figure 7). Second most significant factors were urbanity for the onset of the flowering and nitrogen dioxide levels for the peak and end of the season; other parameters like tree morphometric features as well as soil and other factors were proven not to play a significant role in this setup (Figure 7). 

To additionally estimate the relationship between temperature and the timing of flowering, the cumulative temperature from 1st January until the start of flowering was considered. The relationship (*R*^2^) between the flowering start day and the cumulative minimum temperature is higher than 0.95 in every year and as averaged from all years (Table 2) even though it is shifted in different years, while the relationships of the flowering peak, end, and duration are weaker.

**Figure 8:**
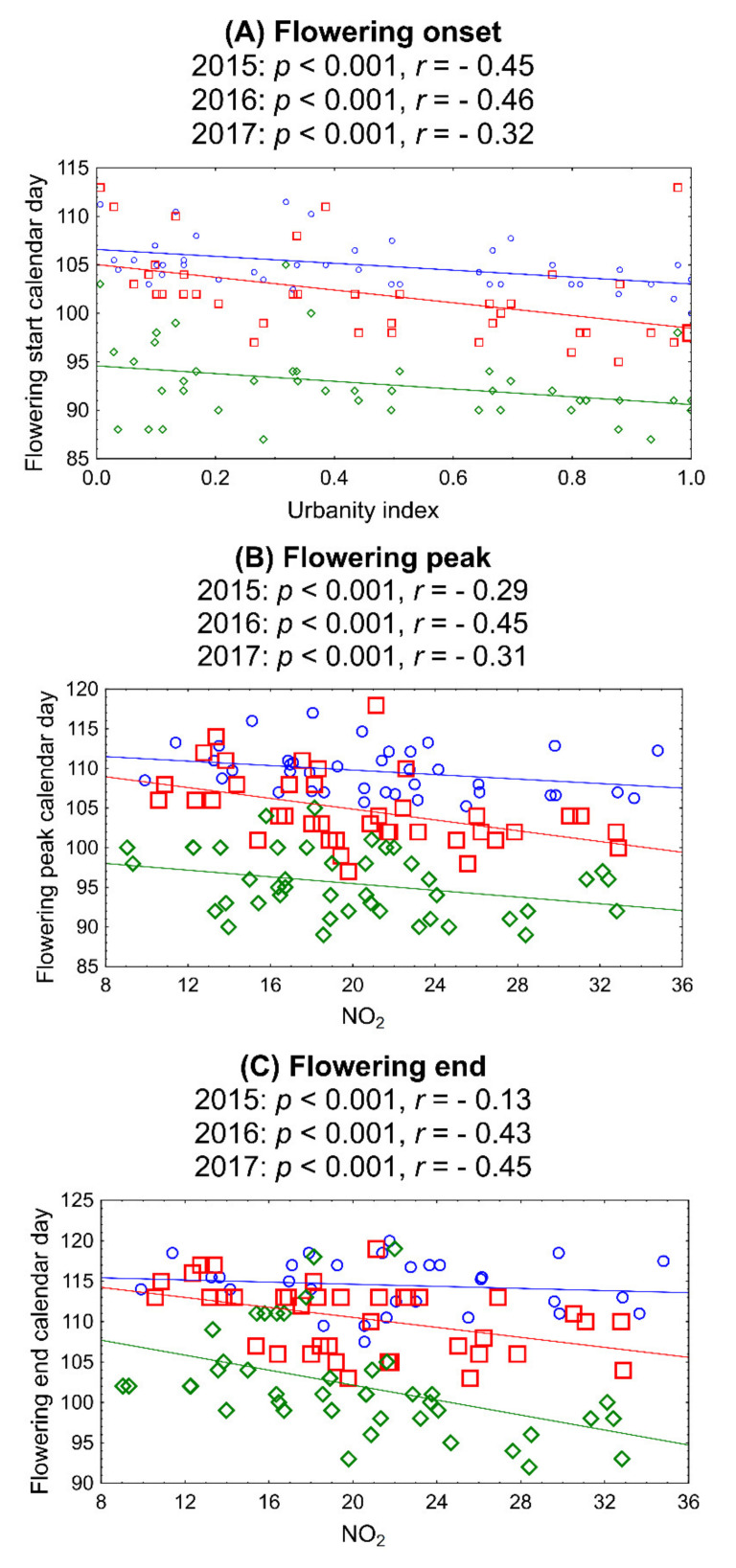
Relationships between flowering phenological traits and environmental parameters in Augsburg, during 2015–2017 (linear regressions, per year, weighted for air temperatures). (**A**) Flowering onset vs. urbanity index, (**B**) flowering peak vs. NO_2_, (**C**) flowering end vs. NO_2_. Blue: year 2015, red: 2016, green: 2017.

Based on the significance in the backwards stepwise ridge regression (Figure 6), the relationships between the phenology traits and the urbanity index and NO_2_ were further plotted and depicted, per year, in Figure 8. The flowering season started earlier in urban environments and peaked and ended earlier in environments with higher NO_2_ levels (*p* < 0.05); the season was shifted rather than prolonged, as the duration gave no significant relationship with either factor (*p* > 0.05). Despite the significance of the relationships in all years, the signal was rather low, with the strongest being consistently for the onset of the flowering season (Figure 8). 

## 4. Discussion

Phenological traits of flowering are known to be sensitive to environmental stress [47]. Hence, in the current study, we explored flowering phenology of a common woody anemophilous plant species, *Betula pendula*, whose pollen comprises a major allergen-trigger in sensitised individuals across the Northern Hemisphere, and we studied its responsive ability under differing environmental regimes. We compared the flowering seasons of the species locally, at the habitats where they occur, with the regional pollen seasons of the respective pollen taxa (*Betula* spp.) to which they belong, so as to search for commensurate changes. As flowering seasons are expected to be reflecting a large proportion of the respective pollen season, we also originally hypothesised the same. Our salient finding here, though, reveals different evidence: indeed, specific environmental parameters, namely air temperature, urbanity, and NO_2_, influence the seasonality of flowering; nonetheless, the relationships between them and with the pollen seasonality are not very strong. This implies that there are hidden additional factors that could be also, synergistically, affecting the system of flowering-pollen seasonality. Such comparisons between the seasons of flowering and pollen, from detailed field observations and from a wide range of habitats are lacking, potentially because of the labour-intensiveness of the methods implicated.

The above mismatch between *Betula pendula* flowering season and the *Betula* spp. pollen season cannot be a methodological artefact, as *B. pendula* is very abundant in the regional vegetation and the flowering phenology was conducted in numerous different individual trees, scattered across the study area. Therefore, we consider them as a representative sample of the regional pollen flora. Here it is evident that even though it is believed that pollen seasons are in general well synchronised with flowering seasons [48], this is not always the case, as this co-occurrence highly depends on a wide range of factors, such as latitude, altitude, and microclimate [18,49,50]. Lack of synchronisation between flowering and pollen seasons can be also due to long-distance transport of pollen, from sources farther away than the locally flowering individuals. Additional spatial analyses, like back trajectory modelling, have already highlighted such pollen atmospheric circulation patterns for the study area [51]. Overall, the shape of the *Betula* pollen season in Augsburg, Germany, seems consistent among years, being short and highly peaked in all years, with usually long tails after the large peak, as also described by Piotrowska [52], who also shows a high seasonal variability between years. This can be partly explained by different *Betula* species, emitting pollen at different times: the Organisation for Economic Cooperation and Development [53] describe a difference of one week in the timing of flowering between *Betula pendula* and *Betula pubescens*, with *Betula pendula* flowering first. 

Flowering is also influenced by a variety of factors, the most important of which have been found to be air temperature, urbanity and NO_2_. This high plasticity was shown before by various researchers for different influencing factors and plant species, for example *Corylus avellana* in Poland [54], different species of *Cupressus* in Spain [55], different species of *Quercus* in Spain [56] and 13 different taxa from Corylaceae, Cupressaceae, Fagaceae, Oleaceae, Pinaceae, and Platanaceae in Greece [47]. It has been extensively documented how important air temperature is on plant phenology [50,55,57], all of the research showcasing that higher temperatures lead to earlier flowering. Importantly, temperatures do not just influence phenology as a temporal variable, but also on a spatial scale, referring to the microenvironment, for example, as an effect of urbanity (urban heat island effect), as has also been reported for the study area of Augsburg as well [33,58].

Moreover, we found here that NO_2_ levels play an important role in the flowering of birch, mainly in its peak and end dates. This highlights that apart from the more generalised and strong effect of factors like air temperature and urbanity, point-source parameters like air pollutants may set an influence. However, this impact is more variable and with increased fluctuations spatially and, thus, affecting mainly the peak and the end of the phenological seasons. Such influences of air quality on plant phenology have been shown for a range of plants and pollutants [59,60,61]. Here we need to point out that the study area refers to a city with a lot of green spaces (Figure 1) and with roughly any exceedances of NO_2_ concentrations (according to the World Health Organization). Therefore, if such comparatively low NO_2_ levels significantly affect the responsive ability of plants, it is considered certain that here we might have a clear underestimation of this impact and further research on more polluted areas in the world need to be conducted.

Overall, there is undoubtedly a huge variety of environmental factors that may be affecting the phenology of flowering, including but not limited to the above-mentioned parameters. Regional studies, based on site-specific measurements, aim to provide insight on the responsive ability of plants under differing environmental regimes. This plasticity (or the lack of it) would reveal the potential effect of climate change on the reproductive output of plants, here namely on flowering seasonality and pollen attributes. In *Betula*, the flowering season is short (yearly average: 10–11 days), so 70–80% of the pollen per individual tree is released in two to three days, which explains the high variability in both seasons, flowering and pollen. Nonetheless, the signal is so robust that it masks out all other effects: the seasons are shifted earlier because of the above-mentioned environmental factors. Noticeably, the seasons here were not prolonged overall, against commonly held information that pollen seasons are extended: to elucidate this, seasons have to be considered not as a whole, but in a detailed, per-taxon manner.

One should keep in mind that even though the study area, Augsburg, has urban structures, and the urban heat island effect has been reported for the city [58], investigation of more regions around Bavaria and across Germany would further elucidate the dynamics of the phenological processes. Avowedly, the differences in Augsburg between urban and rural areas are rather small, concerning temperature, pollutants and further effects of urbanity. A study by Jochner et al. [62] performed in the neighbouring and much more urban city of Munich revealed that indeed temperature does play a significant role, but there it could explain up to 83.7% of the variance in onset dates, whereas in the here presented data, temperature can explain almost half of this variability.

If we consider how much temperature has been increasing, along with urbanisation and air pollutant levels, the current research is highly relevant and timely under the ongoing climatic change. It has been already highlighted that particularly early flowering plant species face dramatic changes in their phenology (among other traits) [49,63,64,65] and especially for wind-pollinated species [63]. This points out the importance of the continuous and long-term biomonitoring of plant phenological traits. From a public health perspective, achieving a high-accuracy prediction of flowering phenology would significantly help to forecast the timing of allergenic pollen seasons so as to efficiently and timely warn allergic individuals.

## 5. Conclusions

Woody anemophilous plants are very responsive to environmental variability. Even under a wide variety of environmental regimes, urban vs. natural, air quality and meteorological parameters, they consistently behave towards the same direction, which is earlier flowering with warmer micro-environment or year. This also implies sensitivity to the ongoing climate change. These findings practically confirm our original hypothesis of high responsiveness of plant flowering to different environmental regimes.

However, when it comes to airborne pollen seasons, they do not necessarily coincide with the flowering seasons. So, questions are raised whether pollen season alone can be used as an optimal bioindicator of climate change. As a vast scientific monitoring network of airborne pollen operates globally for decades now, it is important to assess what part of the actual climate change is expressed in these time series. We propose the setup of a harmonised, cross-sectional and longitudinal long-term study of airborne pollen concentrations across the globe: this would allow clarifications of how environmental variability influences plant responses and also at what stage of the climate change effect we currently find ourselves.

## Figures and Tables

**Table 1:** Parameters characterising the surroundings of the trees.

Parameter	Categorisation
open water bodies (e.g., lake, river)	no/yes
non-biological sealing	no/gravel/concrete/wall/mixed
street	no/yes
sealed areas	no/low (<50%)/high (>50%)
diversity of plants in the herbaceous layer	no/low diversity (agricultural fields)/medium div. (meadow, garden)/high div. (multiple environments)
diversity of trees and shrubs	no/*Betula*/mixed incl. *Betula*/mixed, other/shrubs
shading of the tree	no/low/medium/high
positions of buildings	cardinal directions from the tree

**Table 2:** Relationships of flowering start, peak, and end dates against cumulative temperature, as well as the flowering season length. Coefficients of determination are given, along with the average cumulative value and the standard deviation, in parenthesis; ‘ns’ indicates lack of significant relationship.

Year	Flowering Trait			
	Start	Peak	End	Duration
2015	0.95 (104 ± 2)	0.50 (109 ± 2)	0.17 (115 ± 3)	ns
2016	0.99 (102 ± 4)	0.70 (105 ± 5)	0.51 (110 ± 4)	0.23 (10 ± 3)
2017	0.96 (93 ± 4)	0.72 (95 ± 4)	0.54 (102 ± 6)	ns
Average	0.97 (99 ± 6)	0.85 (102 ± 7)	0.71 (108 ± 7)	ns

## Data Availability

All data are readily available in Excel format and may be provided upon request.
